# Editorial: Insights in endocrinology of aging: 2021

**DOI:** 10.3389/fendo.2022.1066216

**Published:** 2022-11-15

**Authors:** James M. Harper

**Affiliations:** Department of Biological Sciences, Sam Houston State University, Huntsville, TX, United States

**Keywords:** hormones, pregnancy, sleep, metformin, healthspan, longevity

Since the mid-twentieth century, the proportion of elderly adults in the world’s population has steadily increased. Consequently, life events typically associated with youth, for example, pregnancy and childbirth, have become more common in older individuals. Efforts to gain a better understanding of how factors such as pregnancy, altered endocrine profiles and sleep patterns, as well as changes in metabolic function, are influenced by age have become increasingly important, especially with regard to healthspan, an indicator of the quality-of-life at an advanced age. Interventional strategies aimed at extending healthspan, even in the absence of a marked increase in longevity, are needed. In this Research Topic, the interplay of aging, reproductive hormones, and physiological function in humans is examined in addition to a critical review of the putative role of the widely prescribed antidiabetic drug metformin in regulating human longevity and healthspan.

The age at first pregnancy for women has risen largely due to new societal norms that delay childbirth among reproductively competent women. Hence, pregnancies at an advanced maternal age (AMA) have become increasingly common. To address how age affects the steroidomics of pregnancy, Yu et al. collected maternal and fetal cord blood samples, as well as placental tissue from females aged 35 and above (AMA) experiencing pregnancy and from those aged 34 and below. These samples were used to profile the levels of select estrogens, androgens, glucocorticoids, and the mineralocorticoid deoxycorticosterone. Not surprisingly, marked variation in the levels of individual steroids existed among sources (e.g., maternal versus fetal cord blood), but only androgens in maternal blood varied significantly among women of AMA relative to young controls. Moreover, within AMA women the level of these same androgens differed significantly among individuals that experienced pregnancy complications versus those that did not. Although the mechanistic basis for these differences (e.g., the differential activity of CYP enzymes) is unknown, this study has provided significant insight into endocrine mechanisms underlying increased risk of abnormal pregnancy with advancing age.

The cessation of menses (i.e., menopause) is inevitable for women and typically occurs around the fifth decade of life. Due to the dramatic change in circulating hormone levels, especially the loss of estrogen-dependent signaling, multiple health risks have been linked to the onset and progression of menopause. Using a longitudinal dataset collected from 18 – 50 year-old women over almost two decades, Tehrani et al. provide convincing evidence that long-term monitoring of anti-Mullerian hormone (AMH) is a reliable predictor of the age at menopause in this population. Continued improvement in predicting the time of menopause, as well as the onset of other age-related states, has important implications for improving the healthspan of our increasingly aged population.

Meanwhile, a significant long-term risk of menopause is sarcopenia, the loss of muscle mass and the accompanying decline in muscle quality and function. Individuals suffering from sarcopenia demonstrate increased frailty and a reduction in healthspan, regardless of the cause. In the case of menopause, sarcopenia is thought to be largely due to the loss of estrogen-dependent signaling. This has led to hormone replacement therapy (HRT) being advocated to counter the effects of menopause on sarcopenia and other conditions common in postmenopausal women. In their mini-review, Geraci et al. focus on the role of estradiol in the development of sarcopenia and the potential benefits of nutritional and physical interventions coupled with estradiol replacement therapy to counter the negative effects of menopause on muscle function to increase the healthspan of postmenopausal women.

As a complement to the work of Yu et al. demonstrating an association between maternal androgen level and the risk of complications during pregnancy in women of advanced maternal age, Zhao et al. report that the risk of experiencing one or more morbidities allied with a decline in healthspan can be ascertained using serum testosterone level alone or in conjunction with specific genetic factors. Interestingly, these associations were found in both men and women; however, they were sex-specific, and in opposition to serum testosterone levels. Akin to prescribing estradiol replacement therapy for menopausal women, testosterone replacement therapy has widespread clinical use to counter the presumed effects of reduced testosterone in older men, as well as being used in postmenopausal women to counter reductions in sex drive. Given its robust sample size (>290,000 individuals) the results of this study will aid in refining the guidelines currently used for the application of HRT in both men and women.

Pharmaceutical interventions to stave off the effects of aging on functional outcomes (i.e., healthspan), or to simply increase longevity, are desirable due to ease of use and widespread availability. Hormone replacement therapies, as mentioned previously, are excellent examples. The insulin sensitizer metformin has been used to treat individuals with type 2 diabetes mellitus for over 6 decades and has more recently garnered attention as a putative anti-aging drug. In a critical review of the literature, Mohammed et al. posit that evidence for a direct life-extending effect of metformin remains ambiguous but there is strong evidence that metformin can extend healthspan consequent to a reduced rate of early mortality for individuals suffering from various disease states.

Metabolic syndrome is the consequence of altered endocrine function, most notably insulin resistance, and not surprisingly, those suffering from metabolic syndrome also suffer from increased mortality. While inactivity and obesity are established causes of metabolic syndrome, the study by Che et al. has added improper sleep to the list of causal factors involved in the development of metabolic syndrome using data from more than 300,000 individuals. In short, either too little sleep or too much sleep was equally likely to contribute to the development of metabolic syndrome. Fortunately, sleep behavior is amendable, and the promotion of healthy sleep habits would significantly improve human healthspan.

These studies serve to highlight the myriad factors that can contribute to reduced healthspan. Continued study of these factors, and others, will serve us well in developing strategies to mitigate their effects and improve the quality of life in the increasingly elderly population.

## Author contributions

The author confirms being the sole contributor of this work and has approved it for publication.

## Conflict of interest

The author declares that the research was conducted in the absence of any commercial or financial relationships that could be construed as a potential conflict of interest.

## Publisher’s note

All claims expressed in this article are solely those of the authors and do not necessarily represent those of their affiliated organizations, or those of the publisher, the editors and the reviewers. Any product that may be evaluated in this article, or claim that may be made by its manufacturer, is not guaranteed or endorsed by the publisher.

